# Alzheimer’s patients do not show left unilateral spatial neglect but exhibit peripheral inattention and simplification

**DOI:** 10.1590/S1980-57642008DN10400008

**Published:** 2007

**Authors:** Mari Kasai, Junichi Ishizaki, Kenichi Meguro

**Affiliations:** 1 Occupational Therapist, Lecturer, PhD, Department of Geriatric Behavioral Neurology, Tohoku University Graduate School of Medicine, Sendai, Japan.; 2 Associate Professor, PhD, Kobe-Gakuin University, Kobe, Japan.; 3 Professor, MD, PhD, Department of Geriatric Behavioral Neurology, Tohoku University Graduate School of Medicine, Sendai, Japan.

**Keywords:** Rey-Osterrieth complex figure test, Alzheimer’s disease, visual inattention, unilateral spatial neglect, teste da figura complexa de Rey-Osterrieth, teste da bisseção de linhas, doença de Alzheimer, inatenção visual, negligência espacial unilateral

## Abstract

The Rey-Osterrieth Complex Figure Test (RCFT) is widely used to measure visuoperceptual and visuoconstructional skills, while the Line Bisection (LB) test is commonly employed to assess unilateral spatial neglect (USN). Previous studies have suggested that Alzheimer’s disease (AD) patients may suffer from left USN.

**Objectives:**

The purpose of this study was to clarify whether left USN occurs in AD.

**Methods:**

Forty controls, 40 very mild AD patients and 31 mild/moderate AD patients performed both the RCFT copying and the LB test.

**Results:**

The very mild AD and mild/moderate AD groups had lower total RCFT copying scores and also scored lower in the “left” and “detail” categories compared to controls. However, there were no correlations between the left-category score for RCFT and the LB score. Instead, peripheral inattention and simplification patterns were noted.

**Conclusions:**

We found that the RCFT copying test is effective for detecting early AD and suggest that AD patients manifest peripheral inattention and simplification but not left USN.

## Abstract (pt)

O teste da figura complexa de Rey-Osterrieth (TFCR) é muito utilizado para avaliar habilidades vísuo-espaciais e vísuo-construtivas, enquanto o teste de Bisecção de Linhas (TBL) é usualmente empregado para avaliar a presença de negligência espacial unilateral. Estudos anteriores têm sugerido que pacientes com doença de Alzheimer (DA) podem apresentar negligência espacial unilateral esquerda.

**Objetivos:**

O propósito deste estudo foi o de verificar se negligência espacial unilateral esquerda ocorre na DA.

**Métodos:**

Quarenta controles, 40 pacientes com DA muito leve e 31 com DA leve/moderada foram avaliados mediante o TFCR e o TBL.

**Resultados:**

Os pacientes com DA muito leve e com DA leve/moderado tiveram escores mais baixos na cópia do TFCR e também nas categorias “esquerda” e “detalhe” comparados com controles. Entretanto, não houve correlação entre os escores na categoria-esquerda do TFCR e os escores no TBL. Por outro lado, foram notados padrões de inatenção periférica e simplificação.

**Conclusões:**

Nós observamos que a cópia do TFCR é eficaz para detector DA inicial e sugerimos que pacientes com DA manifestam inatenção periférica e simplificação, mas não negligência espacial unilateral esquerda.

Patients with Alzheimer’s disease (AD) have been shown to exhibit unilateral spatial neglect (USN),^1-3^ while Cherrier et al.^3^ reported that AD patients showed left-sided errors or inattention during copying in the Rey-Osterrieth Complex Figure Test (RCFT).^4-6^ Impaired parietal lobe function (i.e., disabilities of visual search and disengagement) has been implicated in left USN, where Ishiai^1^ and Venneri^7^ suggested that some AD patients with left USN have impairment of the right temporal and parietal regions. Ishiai^8^ also reported left USN in 32 mild to moderate AD patients based on the results of a Line Bisection test (LB)^9^ and the RCFT.

Since there have been few reports on AD patients with left USN, the criteria for determining this characteristic in AD are unclear. In Cherrier’s study^3^, the RCFT was divided into six categories: gestalt, detail, right, left, upper and lower, with the number of test items differing in each category. In Ishiai’s study,^8^ the criterion for LB deviation was a rightward error of more than 4 mm on a 200-mm line. However, this deviation may be too small to allow detection of USN, and the criterion might prove too strict for AD patients. Assuming that AD patients have USN, their impairment is likely to be milder than patients with cerebrovascular disorders. Alternatively, impairment in AD might lead to omission, impoverishment or fragmentation,^10^ probably due to visual inattention,^11^ and it is of note that Kaskie et al.^12^ reported visual inattention of very mild AD patients using the Benton Visual Form discrimination test.^13^

We hypothesized that the visuospatial deficit of AD might be due to visual construction disability or peripheral visual inattention, rather than based on left USN. Therefore, the purpose of this study was to determine whether left USN can be detected in AD patients using the RCFT copying and the LB test.

## Methods

### Subjects

The Osaki-Tajiri Project is a community-based study on stroke, dementia, and bed-confinement prevention in the Tajiri district of Osaki. As part of this project, we targeted all residents aged 65 years or older (N=3,207) from November 1998 to March 2001,^14,15^ where 1,654 (51.6%) of these residents agreed to participate in the project. After excluding subjects with cerebrovascular disease and psychiatric illness, 625 subjects were randomly selected. These subjects underwent Clinical Dementia Rating (CDR)^16,17^ and neuropsychological tests including RCFT copying and the LB test.

For the current study, we enrolled 40 healthy elderly subjects (CDR 0), 40 patients with very mild AD (CDR 0.5), and 31 mild/moderate AD patients (CDR 1 and 2). Written informed consent was obtained from all the healthy subjects and from the caregivers of patients with very mild AD and mild/moderate AD. The Ethics Committee of Tajiri approved the study. There were no significant differences in age, educational level and gender among the three groups (see below). Table 1 shows the demographics of the study population.

**Table 1:** Demographics of the study population.

	Healthy (CDR0)	Very mild AD (CDR 0.5)	Mild/moderate AD (CDR 1&2)
N (male / female)	40 (17/23)	40 (16/24)	31 (11/20)
Age, yrs*	81.0 (6.0)	81.5 (6.1)	81.1 (6.3)
Educational level, yrs*	7.8 (2.1)	7.6 (2.0)	8.0 (3.0)

* Mean (standard deviation). There were no significant differences in age (F=0.08, p=0.93), educational level (F=0.25, p=0.78), or gender (χ^2^=0.36, p=0.83) among three groups. Age and educational level: one-way ANOVA; Gender: chi-square test.

### CDR assessments

A clinical team comprising medical doctors (board-certified neurologists and a psychiatrist) and public health nurses determined the CDR stages, independently of the neuropsychological assessment, in the following manner:

1) Before the subjects were interviewed by the medical doctors, public health nurses visited the subjects’ homes to evaluate their daily activities.
2) Observations by family members regarding the subjects’ lives were described in a semi-structured questionnaire. For subjects who lived alone, public health nurses made frequent visits to evaluate their daily lives.
3) The subjects were interviewed by medical doctors to assess episodic memory orientation and judgment.
4) Finally, with reference to the information provided by family members, the subjects’ CDR stages were decided at a joint meeting of the medical doctors and public health nurses.

A reliable Japanese version of the CDR scale had previously been established.^18^ Dementia (CDR 1+) was diagnosed by the DSM-IIIR.^19^ One of the authors (K.M.) was certified as a CDR rater at the Alzheimer’s Disease Research Center Memory & Aging Project, Washington University School of Medicine.

The CDR 0 subjects were community dwelling healthy adults without any cognitive or physical problems. The CDR 0.5 patients showed mild cognitive dysfunction with no apparent problems with daily activities in a community; the CDR 0.5 stage is considered to be a transitional stage between normal aging and dementia. According to Morris et al.,^20,21^ patients with CDR 0.5 already have specific Alzheimer’s disease (AD) pathology; that is, they correspond approximately to very mild AD. Therefore, individuals in the CDR 0.5 and CDR 1+ groups were considered to include possible and probable AD patients, respectively (NINCDS-ADRDA).^22^

### Measurements

Trained clinical psychologists, public health nurses, and occupational therapists administered the RCFT copying and LB tests, while blinded to the CDR assessment.

### RCFT copying

The RCFT requires copying and reproduction of a complex figure, and we have previously reported a large RCFT data set for healthy elderly people living in the same community.^23^ In the current study, two raters independently scored the test according to the method of Lezak^4^ and Meyers.^5^ The complex figure was divided into 18 units, and each unit was scored separately for accuracy and placement. A score of 0, 0.5, 1 or 2 was assigned to each unit, and the unit scores were then summed to obtain the raw score, which therefore ranged from 0 to 36. The mean of the scores determined by the two raters was used as the final score. In cases of an inter-rater difference of three points or more, a third senior rater also scored the test, and the mean of the closest two scores was used as the final score.

### LB test

The LB test is a simple but sensitive method for detecting patients with USN.^9,24^ A test using a line of 120 mm or more was effective in detecting patients with USN.^25^

However, a test using a shorter line of 40 mm or less was not effective, where a cross-over effect occurred.^26,27^ In this study, we used 4 lines: two 120-mm lines and two 200-mm lines on a sheet of paper 210×297 mm in size. The subjects were given the sheet of paper with two sets of four lines, which were placed centrally, and mean deviations from the middle of the line were calculated for the four 120-mm and four 200-mm lines. A plus (+) deviation reflects a “right deviation” from the mid-point, indicating the presence of left USN, and a minus (-) deviation reflects a “left deviation” from the mid–point, indicating the presence of right USN. The average values of 4 assessments of both 120-mm lines and 200-mm lines are shown in Analysis 1 (see below) and those of 200-mm lines were used for Analysis 3 (see below).

### Analyses

In all statistical analyses, differences were considered significant at p<0.05.

#### Analysis 1: Total and sub-category scores for RCFT copying, and LB test scores

Scores were compared among the three groups (healthy, very mild AD, and mild/moderate AD groups) using multiple ANOVAs.

#### Analysis 2: Reversed figures on the RCFT

**Figure 1:**
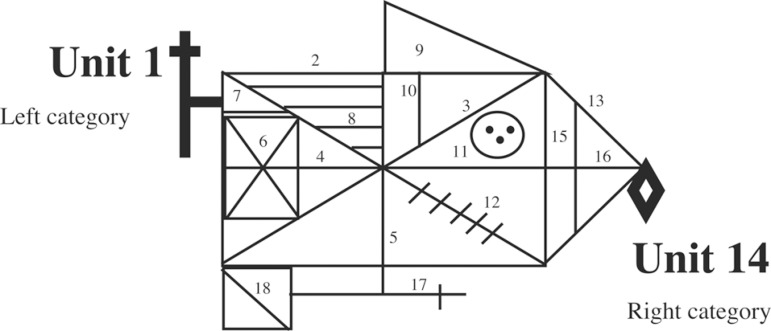
Illustrations of the RCFT figures emphasizing the unit 1 and 14.

**Figure 2:**
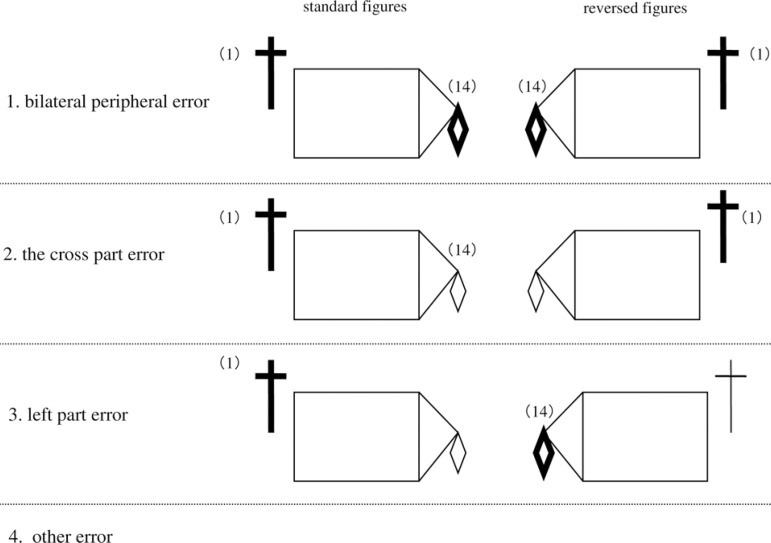
Four error patterns on the RCFT figures of the standard and reversed versions.

To determine whether impaired drawing on the left part of the RCFT was due to left USN, or to the specificity of the “cross” part, or to peripheral inattention, copying of the standard RCFT stimulus figure and a reversed figure was compared. The reversed figure was made by reversing the left and right sides of the standard figure. Four error patterns were analyzed: 1) the “bilateral peripheral error”, i.e., errors in unit 1 (left category) and unit 14 (right category) of the reversed and standard figures (see Figure 1 for illustrations of the RCFT figure emphasizing units 1 and 14); 2) the “cross part error” for the reversed and standard figures; 3) the “left part error” for the reversed and standard figures; and 4) “other error” patterns, including partial versions of the first three patterns, such as, for example, a right-category error on the standard figure, but no error on the reversed figure. Figure 2 illustrates the four error patterns. Chi-square tests were used for analysis of the data.

#### Analysis 3: Left cross (unit 1) scores in RCFT copying and LB test deviations

To examine the presence of left USN, we analyzed the relationship between the left cross score (unit 1) and deviations in the LB test in the three groups, using Spearman’s rank-correlation coefficient and Fisher’s exact probability test. The LB was judged using Ishiai’s criteria, according to which, 4 mm and over is considered to be a significant deviation.^8^

## Results

### Analysis 1

There were significant differences in total RCFT scores among the three groups where step-by-step deterioration in these scores occurred from healthy subjects to very mild AD patients and to mild/moderate AD patients (Table 2). In Cherrier’s six categories of RCFT copying, there were also significant differences between the healthy subjects and the mild/moderate AD groups, except for the upper category. Scores on the detail and left categories for the very mild AD group were significantly lower than the respective scores for the healthy group. There were no significant differences among the three groups for deviation from the 120-mm and 200-mm lines in the LB test.

**Table 2:** The scores for RCFT copying, the LB and Cherrier's six categories classification of groups of healthy, very mild AD, and mild/moderate AD subjects.

Domais	Full score	Healthy			Very mild AD			Mil/moderate AD			*F* values	*p* values
		n	(CDR0)		n	(CDR 0.5)		n	(CDR 1&2)			
RCFT												
Copying total score	36.0	40	30.3 (4.9)		40	26.1 (8.6)^a^		31	21.5 (9.7)^ab^		11.0	0.00**
Cherrier's six categories												
Gestalt (unit 2,3,4,5,13,16)	2.0		1.9 (0.2)			1.7 (0.5)			1.4 (0.6)^ab^		10.79	0.00**
Detail (unit 6,7,8,10,11,12,15)	2.0		1.6 (0.4)			1.3 (0.5)^a^			1.0 (0.6)^a^		10.72	0.00**
Right (unit 14)	2.0		1.7 (0.6)			1.5 (0.7)			1.2 (0.9)^a^		5.09	0.01*
Left (unit 1)	2.0		1.6 (0.6)			1.2 (0.8)^a^			0.8 (0.8)^a^		10.16	0.00*
Upper (unit 9)	2.0		1.8 (0.5)			1.6 (0.8)			1.4 (0.8)		2.74	0.07
Lower (unit 17,18)	2.0		1.6 (0.5)			1.4 (0.7)			1.2 (0.7)^a^		4.35	0.02*
LB (120 mm)	60.0	40	0 (2.3)		40	-0.2 (3.4)		31	-0.4 (3.5)		0.15	0.86
LB (200 mm)	100.0	40	-0.2 (4.2)		40	-0.5 (4.3)		31	-1 (4.6)		0.29	0.75

Means shown (SD), F values and p values (covariance effects). Significant differences among the three groups were shown on ANOVAs (*p<0.05, **p<0.001). On Tukey-Kramer post hoc tests, "a" was significantly lower than healthy, "b" was significantly lower than very mild AD. Using Cherrier's six categories classification (Neuropsychiatry Neuropsychol Behav Neurol, 1999;12:95-101). On the LB, the mean of deviations from the middle for 120 mm or 200 mm lines were calculated. CDR: Clinical Dementia Rating; RCFT: Rey-Osterrieth Complex Figure test; LB: Line Bisection test; AD: Alzheimer's disease.

**Figure 3:**
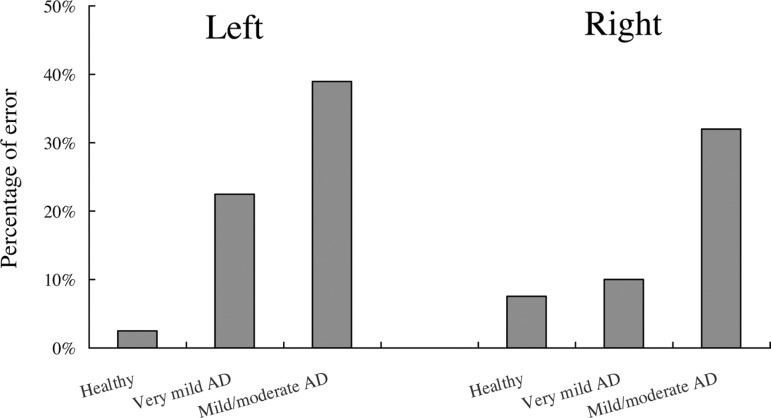
Percentage of subjects who showed “no drawing” errors for the left and right categories of the RCFT.

The percentage of subjects whose left or right scores were assessed as “no drawing” are shown in Figure 3. In the left category, the percentage of subjects with a “no drawing” error was 3% in the healthy group, 23% in the very mild AD group, and 39% in the mild/moderate AD group. In the right category, these percentages were 8% in the healthy group, 10% in the very mild AD group, and 32% in the mild/moderate AD group. These data showed a significant left-right effect among the three groups (chi-square; χ^2^=6.23, p=0.04).

### Analysis 2

Twenty-six mild/moderate AD patients were able to copy the standard and reversed figures in the RCFT. The number of patients who showed bilateral peripheral errors for the standard and reversed figures were 9/15 and 9/15, respectively, and the number of patients who had a cross-part error for the standard and reversed figures were 5/15 and 5/15, respectively. The mild/moderate AD patients with a left-part error on the standard and reversed figures were 1/15 and 1/15, respectively, and other error patterns for the standard and reversed figures were observed for 11/15 and 11/15 patients, respectively. There was a significant difference among the four error patterns (χ^2^ =9.08, p=0.03, by chi-square test).

### Analysis 3

**Figure 4:**
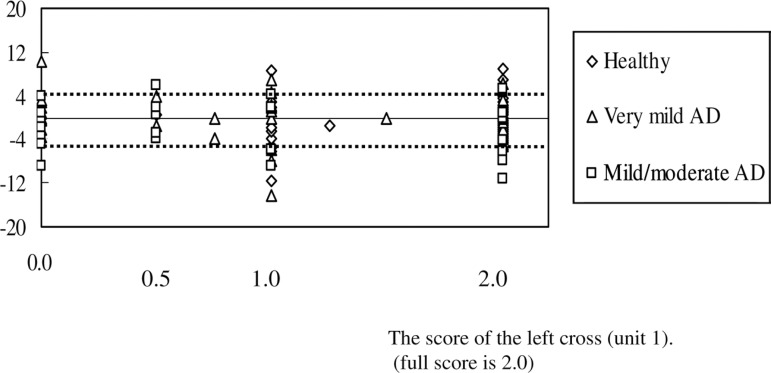
Deviations on the LB test and “left cross” scores for unit 1 in RCFT copying for healthy subjects, very mild AD patients, and mild/moderate AD patients.

Figure 4 shows the left cross (unit 1) scores in RCFT copying as well as deviations on the LB test. Possible left cross scores are 0.0, 0.5, 1.0, and 2.0, where “2.0” is the highest score. There were no significant correlations between the left category on RCFT copying and the LB score in the three groups (r=0.20, p=0.10; r=-0.21, p=0.09; r=0.04, p=0.41 for healthy subjects, very mild AD patients, and mild/moderate AD patients, respectively, based on Spearman rank-correlation coefficient). Using Ishiai’s LB criteria (deviation of 4 mm and over) (Ishiai, 2000), 14 out of 31 mild/moderate AD patients had left (n=5) or right (n=9) USN. There was no significant difference between patients with or without left cross, and left or right USN on the Fisher’s exact probability test (p=0.40)

## Discussion

We did not find a relationship between the pattern of left USN in RCFT copying and LB deviations in AD patients. Therefore, our results suggest that AD patients do not show left USN, but do exhibit peripheral inattention and simplification of construction.

There were no significant differences in age, educational level and gender among the three subject groups. However, our group of AD patients was small, since only 31 AD patients enrolled as participants in the project and subsequently completed the neuropsychological tests. Examination of a larger sample of AD patients is needed to characterize features of visual inattention. However, the tests used in our patient population are reliable. The RCFT^4^ and the LB^9^ are standardized assessment tools, and we tested these methods twice for a few AD patients and confirmed good test-retest reliability (data not shown).

Scores for RCFT copying were significantly lower for the mild/moderate AD group compared to the healthy group, confirming the visuospatial deficit of AD patients found in previous studies.^3,10,12^ For the left category, the very mild AD group had larger error scores than the healthy group, suggesting possible development of visual inattention in very mild AD patients. The characteristic feature of visual constructional ability deficit in AD patients was poorer drawing skills, compared to healthy elderly subjects where this has been defined as “simplification”.^28^

To account for the visual inattention of very mild AD patients, what neural bases were considered? Generally, the right parietal lobe dominates the visual attention function, but the temporal sequence of AD pathology has also been established,^29^, i.e., there is early involvement of the hippocampus followed by the temporal lobes with the parietal lobes becoming affected later. Thus, we should regard the disability as a kind of neural network disorder. The functional decline in the parietal association area is also shown simultaneously with damage to the medial temporal cortex in very mild AD.^30,31^ Although pathological changes do not emerge at the parietal cortex in the early stage,^29^ medial temporal damage is considered to cause functional decline in this area. This notion is supported by an animal study.^32^ We consider the visual attention function to be based on a network, and not as being localized only in this area. We believe that the underlying mechanisms of visual attention disability in very mild AD may not only reflect a deficit due to parietal damage, but also reflect a functional disconnection in the neural network supporting the attention system.

Scores for copying of the reversed and standard figures on the RCFT showed that AD patients had a larger number of errors on the peripheral parts (errors of both left and right categories), compared to other errors patterns. There was a significant difference in the degree of difficulty between the left and right categories on the RCFT. Overall, the results suggest that AD patients might have “peripheral inattention”. However, this conclusion requires further confirmation using a larger sample size.

Ishiai et al. (2000) reported that 25% of AD patients exhibited a left USN error pattern on the LB. We found that 16% of the AD patients (5 of 31) showed left USN on the LB based on Ishiai criteria, and that 29% of the AD patients (9 of 31) showed right USN. However, there were no significant correlations of the left category score in RCFT copying with the LB score for any of the three groups in our study. AD patients may have deficits of “disengaging attention” or “visual exploration”,^2,3^ which also include clumsiness of visual exploration and difficulty in focusing all their attention on a line in the LB test. These characteristics may lead to development of symptoms of “peripheral inattention” in AD patients, which are not limited to left and right symptoms. Moreover, AD patients showed a deficit of visual constructional ability, and have been shown to draw figures poorly and simply; i.e., “simplification”.^28^ As described above, even when AD patients show a left or right deviation on the LB, similar neglect is not observed in a drawing task such as RCFT copying. These data do not support a conclusion of USN in AD patients based on deviation in the LB test. Furthermore, the LB may not be an appropriate test to detect left USN for AD patients, because such patients display “peripheral inattention” and “simplification”. We are performing further studies to explore these conclusions.

In conclusion, AD patients showed pervasive visuospatial deficit. Previous studies have reported that AD patients may have left USN, but our study suggests that AD patients show peripheral inattention and simplification of construction, but not left USN.
